# Defining NELF-E RNA Binding in HIV-1 and Promoter-Proximal Pause Regions

**DOI:** 10.1371/journal.pgen.1004090

**Published:** 2014-01-16

**Authors:** John M. Pagano, Hojoong Kwak, Colin T. Waters, Rebekka O. Sprouse, Brian S. White, Abdullah Ozer, Kylan Szeto, David Shalloway, Harold G. Craighead, John T. Lis

**Affiliations:** 1 Department of Molecular Biology and Genetics, Cornell University, Ithaca, New York, United States of America; 2 Division of Oncology, Department of Internal Medicine, Washington University School of Medicine, St Louis, Missouri, United States of America; 3 School of Applied and Engineering Physics, Cornell University, Ithaca, New York, United States of America; Friedrich Miescher Institute for Biomedical Research, Switzerland

## Abstract

The four-subunit Negative Elongation Factor (NELF) is a major regulator of RNA Polymerase II (Pol II) pausing. The subunit NELF-E contains a conserved RNA Recognition Motif (RRM) and is proposed to facilitate Poll II pausing through its association with nascent transcribed RNA. However, conflicting ideas have emerged for the function of its RNA binding activity. Here, we use *in vitro* selection strategies and quantitative biochemistry to identify and characterize the consensus NELF-E binding element (NBE) that is required for sequence specific RNA recognition (NBE: CUGAGGA(U) for *Drosophila*). An NBE-like element is present within the loop region of the transactivation-response element (TAR) of HIV-1 RNA, a known regulatory target of human NELF-E. The NBE is required for high affinity binding, as opposed to the lower stem of TAR, as previously claimed. We also identify a non-conserved region within the RRM that contributes to the RNA recognition of *Drosophila* NELF-E. To understand the broader functional relevance of NBEs, we analyzed promoter-proximal regions genome-wide in *Drosophila* and show that the NBE is enriched +20 to +30 nucleotides downstream of the transcription start site. Consistent with the role of NELF in pausing, we observe a significant increase in NBEs among paused genes compared to non-paused genes. In addition to these observations, SELEX with nuclear run-on RNA enrich for NBE-like sequences. Together, these results describe the RNA binding behavior of NELF-E and supports a biological role for NELF-E in promoter-proximal pausing of both HIV-1 and cellular genes.

## Author Summary

RNA polymerase II (Pol II) is a molecular machine that is responsible for transcribing all protein coding genes in the eukaryotic genome. Transcription by Pol II is a highly regulated process consisting of several rate-limiting steps. During transcription elongation, a number of transcription factors are essential to modulate Pol II activity. One of these factors is the Negative Elongation Factor (NELF), and it plays a major role in promoter-proximal pausing, a widespread phenomenon during early transcription elongation. NELF-E, a protein subunit of the NELF complex contains a conserved RNA binding domain that is thought to regulate transcription through its interaction with newly transcribed RNA made by Pol II. However, the function of the RNA binding activity of NELF-E remains unresolved due to prior conflicting studies. Here, we clarify the RNA binding properties of NELF-E and provide insight into how this protein might facilitate promoter-proximal pausing of Pol II in transcription. Moreover, we identify the precise region of NELF-E binding in one of its known regulatory targets, HIV-1. Taken together, the results presented indicate a dynamic interplay between NELF and specific RNA sequences around the promoter pause region to modulate early transcription elongation.

## Introduction

RNA polymerase II (Pol II) is a molecular machine responsible for transcribing all protein coding genes in the eukaryotic genome in a highly regulated multistep process. With the help of specific and general transcription factors, it binds to promoters, rapidly initiates transcription, transcribes approximately 20–60 nucleotides of nascent RNA, and then can pause before entering productive elongation [1], [2]. Recent genome-wide studies have demonstrated that promoter-proximal pausing is a frequently observed feature of metazoan genes and a major point of regulation [3]–[5].

Three protein complexes have a major role in Pol II pausing. Two of these, NELF (Negative elongation factor) and DSIF [DRB (5,6-dichloro-1-b-D-ribofuranosylbenzimidazole) sensitivity inducing factor], form a stable complex with Pol II and inhibit its elongation shortly after initiation. In contrast, P-TEFb (Positive transcription elongation factor b), a complex of CDK9 kinase and CyclinT, overcomes the influence of these factors and promotes the release of Pol II into productive elongation [6]–[9]. Experimental evidence indicates that P-TEFb phosphorylates NELF, DSIF, and the C-terminal domain (CTD) of Pol II and that one or more of these modifications alleviate the pause [10]–[12].

Several ChIP-chip and ChIP-seq experiments have revealed that these pausing factors co-occupy promoter-proximal regions of active genes where Pol II also accumulates [3], [13]–[15]. Composite profiles of Pol II demonstrate an overall decrease in promoter occupancy after depleting cells of NELF or DSIF subunits. In contrast, a marked increase in promoter-proximal Pol II occupancy is seen after treating cells with a P-TEFb inhibitor [3], [14], [16]–[18]. In agreement with these observations, knockdown of NELF in *Drosophila* S2 cells leads to a decrease in Pol II density in promoter regions relative to Pol II occupancy in gene bodies [16], [17].

NELF consists of four protein subunits (NELF-A, NELF-B, NELF-C/D, and NELF-E) [7]. NELF-E contains a canonical *βαββαβ* RNA recognition motif (RRM) that is essential for its ability to bind RNA and inhibit elongation *in vitro* [19]. In human cells, the absence of NELF-E abolishes the ability of NELF to repress elongation. This suggests that NELF-E plays a role in the pausing mechanism [20]. One prevailing hypothesis is that NELF-E RNA binding enables NELF to stabilize paused Poll II as the nascent RNA exits the polymerase [19], [20]. However, a recent *in vitro* cross-linking study by Gilmour and coworkers suggested that RNA binding by *Drosophila* NELF-E may not be involved in promoter-proximal pausing, but instead may interact with longer nascent transcripts at a location further downstream [21]. While they show that NELF and DSIF are required to inhibit elongation, they did not identify a NELF/RNA contact among short nascent RNAs that are associated with promoter-proximal paused Pol II. It is possible, however, that the template used in this study lacks a specificity determinant required for an interaction. Therefore, the function for the RNA binding activity of NELF-E remains unresolved.

Studies investigating the regulation of HIV-1 transcription implicate how NELF-E functions [22]. HIV-1 proviral expression is regulated at the level of early elongation, and the leading model suggests that NELF-E binds to the double stranded portion of the RNA transactivation response (TAR) element found between +1 and +59 nucleotides downstream from the transcription start site where Pol II is paused. P-TEFb and the transactivator protein Tat then bind to the TAR element, NELF dissociates, and paused Pol II is then released into productive elongation [23]. Qualitative binding experiments suggest that NELF-E binds to the lower stem region of TAR RNA [12], [19]. In addition, NMR studies have solved the structure of the RRM domain of NELF-E [24], [25]. This work also used fluorescence equilibrium titrations to test its interaction to single and double stranded RNA fragments of the lower stem of TAR. These experiments measured binding affinities in the µM range; however, the precise binding region in TAR RNA was unable to be determined.

Here, we characterize the RNA binding specificity of NELF-E and attempt to clarify its role in promoter-proximal pausing. We demonstrate that NELF-E is capable of binding to RNA with high affinity and specificity. Moreover, we define the NELF-E binding element (NBE) for both *Drosophila* and human NELF-E (dNELF-E and hNELF-E, respectively) and identify the presence of an NBE within TAR RNA, which is located in a different region than previously thought to be bound by NELF-E. Finally, we found that NBEs are enriched at promoter-proximal pause regions in the *Drosophila* genome. This implies a functional role for NELF-E RNA binding in Pol II pausing.

## Results

### Determination of the NELF-E Binding Element (NBE) from selected RNA aptamers

**Figure 1:**
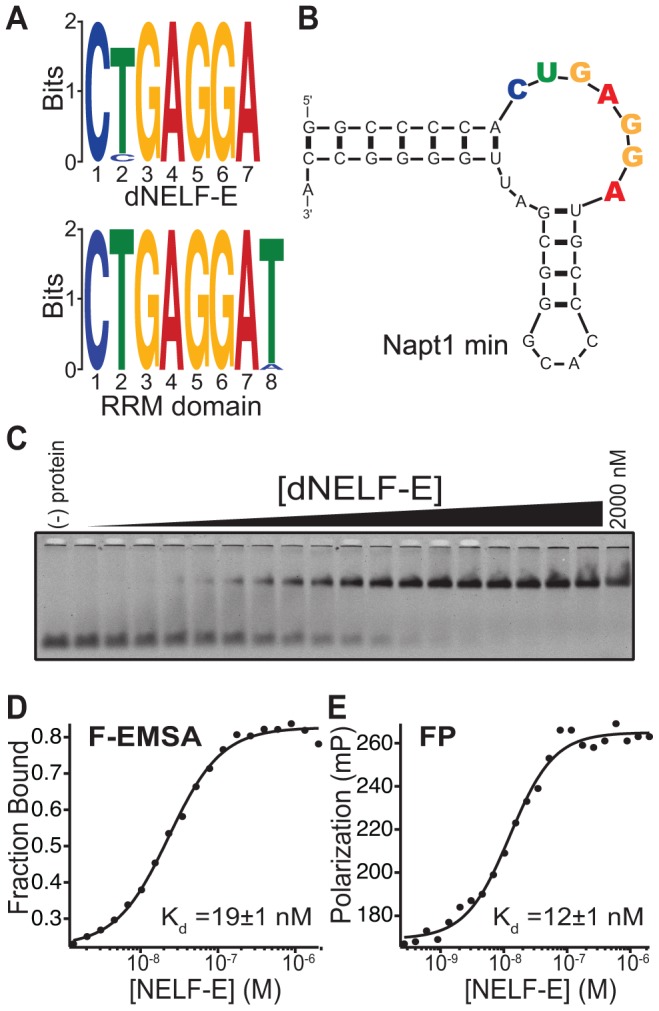
Identification of the NELF-E Binding Element within high affinity aptamers. (a) MEME analysis of the top 3,000 unique clustered sequencing reads from a SELEX experiment of dNELF-E or its RRM domain. The sequence logo derived is shown for both proteins. (b) Secondary structure of Napt1min RNA aptamer. An additional GC base pair was added to the end of the aptamer (see Materials and Methods). The consensus sequence is highlighted with coloring that corresponds to the sequence logo. (c) Full length dNELF-E binds to Napt1min with high affinity. Shown is a representative fluorescence electrophoretic mobility shift assay (F-EMSA) with increasing concentrations of dNELF-E protein from 1.4 nM up to 2 µM and a fixed concentration of fluorescently labeled aptamer. (d) A plot of the fraction of bound Napt1min against protein concentration is presented for the gel in panel (c) and fit to the Hill equation. The equilibrium dissociation constant (K_d_) is shown in the graph and the error represents the standard deviation of the uncertainty of the fit. (e) A plot of fluorescence polarization of the same binding experiment and its measured K_d_ and fit error are presented. Raw polarization values are given in units of milipolarization (mP).

No published studies have investigated the nucleotide specificity of *Drosophila* NELF-E. To identify the sequence specificity of dNELF-E, a microcolumn-based SELEX (Systematic Evolution of Ligands by Exponential Enrichment) experiment was performed with full-length dNELF-E or its RRM domain [26]. The RNA library (>5×10^15^ unique molecules) used contained a 70-nucleotide randomized region flanked by two constant regions that allowed for amplification of selected RNAs and *in vitro* transcription to generate subsequent aptamer pools. This affinity-based approach utilized modular, custom-made microcolumns that permit high-efficiency selection of aptamers by exploiting optimal fluidic parameters [26]. Microcolumns containing protein-bound resin were subjected to six cycles of SELEX, and the resulting pools were sequenced by the high-throughput Illumina Hi-Seq platform to identify putative target-binding aptamer sequences. Approximately 2–4 million sequence reads were obtained for each pool from cycles 4 and 6. After clustering to identify unique sequences, the top 3,000 sequences with the highest multiplicity in pool 6 were analyzed using MEME (Multiple EM for Motif Elicitation), a computational tool that searches for repeated, ungapped sequence patterns from a list of DNA sequences [27], [28]. A highly conserved motif was present within 1,049 out of 3,000 sequences selected for binding to full-length NELF-E and 1,362 of 3,000 sequences for binding to its RRM domain (Figure 1a). These motifs are nearly identical for both proteins and define the NELF-E binding element (NBE) for dNELF-E and its RRM domain as CUGAGGA(U). Examination of the pool 6 sequencing results suggests that the more conserved 3′ position in the NBE from the RRM domain selection is due to faster convergence of NBE containing sequences during earlier SELEX cycles (unpublished data).

Analysis of the most enriched RNA aptamers containing an NBE revealed a common secondary structure consisting of a putative non-canonical kink-turn (K-turn) (Figure S1) [29]. K-turn structures have an asymmetric internal loop that causes a sharp bend between two helical regions. The 3′ end of this loop is typically flanked by a GA/AG Hoogsteen-Sugar edge platform [30]. The NBE is located in the internal loop of the K-turn among candidate aptamers (Figure S1, Figure 1b). A truncated version of the most abundant candidate aptamer, Napt1min, is shown in Figure 1b.

Two approaches were used to quantitatively measure the equilibrium dissociation constant (K_d_) of dNELF-E binding to Napt1min: a fluorescence electrophoretic mobility shift assay (F-EMSA) and a fluorescence polarization (FP) assay, each relying on different physical properties of the protein/RNA complex [31]. Each assay revealed that dNELF-E binds with high affinity to Napt1min (K_d_; F-EMSA = 44±22 nM and FP = 21±7 nM) (Fig. 1c–e; Table 1). Moreover, two other NBE-containing aptamers tested bound with similar high affinity (Figure S2, Table 1, unpublished data). The binding constants measured by F-EMSA and FP were (unless otherwise noted) typically within two-fold of each other, supporting confidence in the measured values.

**Table 1:** NELF-E binding affinity for RNA targets.

Protein	RNA	Sequence	*n*	F-EMSA K_d_ [nM]	FP K_d_ [nM]
**dNELF-E**	NApt1min	GGCCCCACUGAGGAUGCCCACGGGCGAUUGGGGCCA	3	44±22	21±7
	NApt25min	GGUCUCCAACUGAGGAUACCGCUCGAGGAAGCGAGUGGCGAUUUGGAGACCU	3	53±9	30±2
	NApt1+hairpin	GGCCCCACUGAGGAUGCCCACGGGCGUCCUCAGUGGGGCCA	3	810±50	470±170
	NApt1(3G:Amut)	GGCCCCACUAAAAAUGCCCACGGGCGAUUGGGGCCA	1	>2000	ND
	NApt1-Δstem	GGGGACUGAGGAGCAACACGGGCGAUUGGGGCCA	3	205±20	270±130
	NApt1NBEmut	GGCCCCAUCAAAGAUGCCCACGGGCGAUUGGGGCCA	2	880±170	ND
	HIV-1 TAR	GGUCUCUCUGGUUAGACCAGAUCUGAGCCUGGGAGCUCUCUGGCUAACUAGGGAACC	3	350±35	>130
	HIV-1 TAR+A	GGUCUCUCUGGUUAGACCAGAUCUGAGCCUGAGGAGCUCUCUGGCUAACUAGGGAACC	3	59±2	82±1
	HIV-1 TAR-ΔhNBE	5′GGUCUCUCUGGUUAGACCAGAUCUGAGC3′/3′CCAAGGGAUCAAUCGGUCUCUCG5′	3	>2000	ND
**hNELF-E**	NApt1min	GGCCCCACUGAGGAUGCCCACGGGCGAUUGGGGCCA	3	420±90	140±10
	HIV-1 TAR	GGUCUCUCUGGUUAGACCAGAUCUGAGCCUGGGAGCUCUCUGGCUAACUAGGGAACC	3	300±20	200±10
	HIV-1 TAR+A	GGUCUCUCUGGUUAGACCAGAUCUGAGCCUGAGGAGCUCUCUGGCUAACUAGGGAACC	3	250±20	250±20
	HIV-1 TAR-ΔhNBE	5′GGUCUCUCUGGUUAGACCAGAUCUGAGC3′/3′CCAAGGGAUCAAUCGGUCUCUCG5′	3	>2000	ND
**dNELF-E (mut)**	NApt1min	GGCCCCACUGAGGAUGCCCACGGGCGAUUGGGGCCA	2	350±50	95±6
	HIV-1 TAR	GGUCUCUCUGGUUAGACCAGAUCUGAGCCUGGGAGCUCUCUGGCUAACUAGGGAACC	2	270±10	130±10
	HIV-1 TAR+A	GGUCUCUCUGGUUAGACCAGAUCUGAGCCUGAGGAGCUCUCUGGCUAACUAGGGAACC	2	280±30	120±10
**hNELF-E (mut)**	HIV-1 TAR	GGUCUCUCUGGUUAGACCAGAUCUGAGCCUGGGAGCUCUCUGGCUAACUAGGGAACC	3	258±17	153±7
	HIV-1 TAR+A	GGUCUCUCUGGUUAGACCAGAUCUGAGCCUGAGGAGCUCUCUGGCUAACUAGGGAACC	3	233±40	170±5

Values given are the average K_d_ ± s.d. for *n* independent replicates. K_d_ values determined by FP and EMSA were statistically different (p<0.01) only for HIV-1 TAR+A RNA.

### Requirements for *Drosophila* NELF-E RNA binding

**Figure 2:**
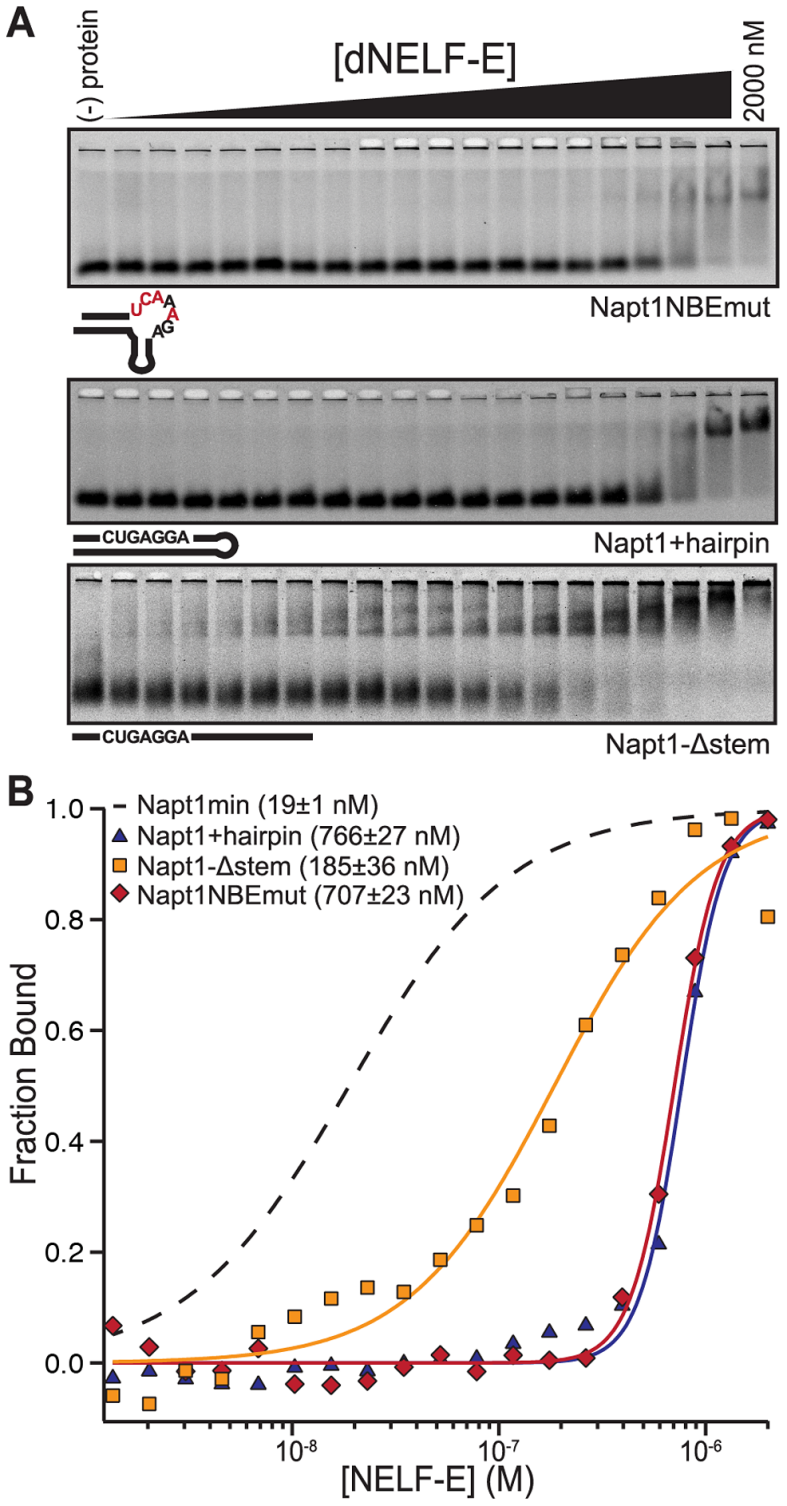
The NBE is necessary and sufficient for dNELF-E binding. (a) A representative F-EMSA of full length dNELF-E binding to Napt1NBEmut RNA, Napt1+hairpin, or Napt1-Δstem. Below each gel is a visual representation of each sequence tested. Mutations made in the NBE are colored red. (b) A normalized plot of fraction bound for each RNA sequence tested in (a). The data and fit are annotated in the graph to indicate measured K_d_ and fit error. For comparison, the fit of dNELF-E binding to Napt1min is shown as a dashed line.

As discussed above, the majority of aptamers selected have the conserved NBE motif and putative K-turn. To assess the contribution that these features have on dNELF-E RNA binding, we generated a variety of Napt1min mutants and tested them for dNELF-E binding. To test the significance of the NBE within Napt1min, a mutant was generated in which four nucleotides within the NBE were changed, but the predicted secondary structure was kept intact (Napt1NBEmut). The binding affinity of dNELF-E to Napt1NBEmut is much weaker (K_d_; F-EMSA = 880±170 nM and FP>2000 nM), demonstrating the importance of the NBE (Figure 2,Table 1).

To determine if binding requires that the NBE is accessible in a single-stranded region, dNELF-E was tested for binding to a Napt1min variant that forms a perfect hairpin by complementary base pairing with the NBE sequence (Napt1+hairpin; Figure 2a). The binding affinity between dNELF-E and this variant is substantially weaker (K_d_; F-EMSA = 810±50 nM and FP = 470±170 nM) compared to that of Napt1min (Figure 2, Table 1). This suggests that an NBE located in dsRNA cannot effectively bind dNELF-E.

Next, to test if dNELF-E requires the putative K-turn structure for high affinity binding, Napt1min was mutated to generate an RNA sequence that has no predicted secondary structure, but still contained the NBE (Napt1-Δstem; Figure 2a). Interestingly, this putatively unstructured sequence is still able to bind dNELF-E with moderate affinity (K_d_; F-EMSA = 205±20 nM and FP = 270±130 nM) compared to the parent minimal aptamer Napt1min (Figure 2, Table 1). This indicates that the putative K-turn present in selected aptamers contributes to dNELF-E binding but is not essential for the interaction. From this group of Napt1min mutants, we conclude that the NBE is necessary and sufficient for RNA binding to dNELF-E so long as it is accessible as single-stranded RNA.

### Both human and *Drosophila* NELF-E bind specifically to the NBE present in HIV-1 TAR RNA

**Figure 3:**
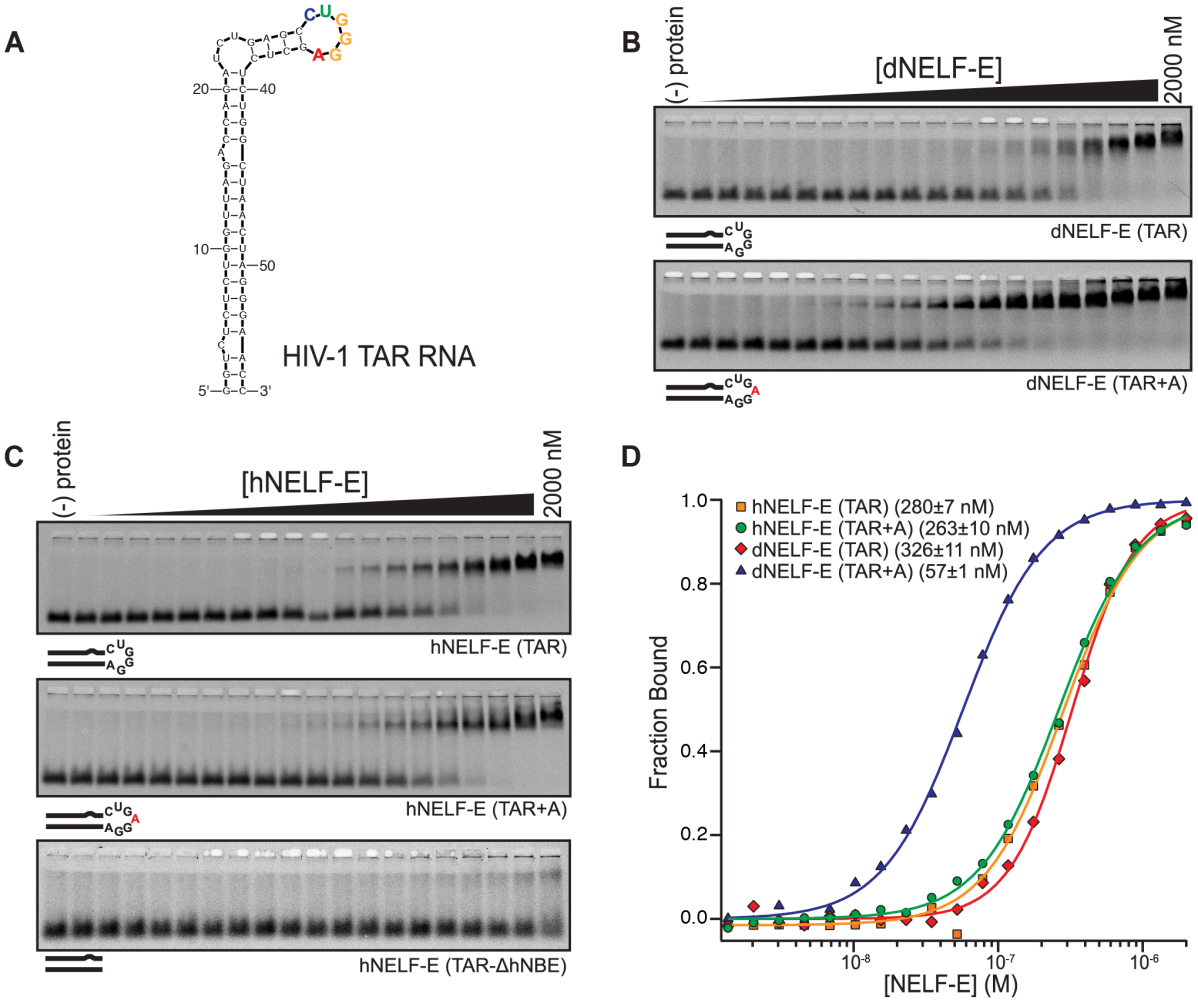
Human and *Drosophila* NELF-E bind specifically to HIV-1 TAR RNA. (a) A secondary structure of HIV-1 TAR RNA. The predicted NBE is colored according to the sequence logo shown in Figure 1a. (b) A representative F-EMSA of full length dNELF-E binding to TAR and TAR+A is shown. Below each gel is a visual representation of the RNAs tested. Mutations are indicated in red. (c) As described in (b), a representative F-EMSA of full length hNELF-E to TAR, TAR+A, or TAR-ΔhNBE are shown. (d) A normalized plot and fit of fraction bound RNA for experiments shown in (b) and (c). The binding constant and fit standard error for each experiment is included next to its label.

The NELF-E RRM is conserved between *Drosophila* and humans, but we were surprised that the reported hNELF-E target, HIV-1 TAR RNA, bore no structural resemblance to our aptamers. HIV-1 TAR RNA forms a highly stable hairpin structure (Figure 3a) that includes a three nucleotide bulge (UCU) that is bound by HIV-1 TAT, and a stem-loop that is bound specifically by Cyclin T1, a subunit of P-TEFb [32]. Previous reports suggested that the hNELF-E RRM binds to the lower stem region of TAR with low specificity and affinity (K_d_>2 µM) [12], [25]. We find that the *Drosophila* homolog, dNELF-E, binds specifically and with high affinity to its RNA targets. Interestingly, a closer examination of the TAR sequence reveals the sequence CUGGGA within the loop region, which is very similar to the NBE sequence CUGAGGA found in Napt1min.

To assess whether dNELF-E is able to bind to TAR RNA, we performed quantitative binding experiments. We found that dNELF-E does indeed bind to TAR, although somewhat weaker than it binds Napt1min (K_d_; F-EMSA = 350±40 nM and FP = 130±10 nM) (Figure 3b,d). Since dNELF-E binds tighter to Napt1min, we examined if it would bind tighter to the TAR element containing the same NBE that was identified by SELEX. To do this, a single adenosine was inserted into the loop region to make an NBE site within the stem loop (TAR+A) and this RNA was tested for binding. Remarkably, this single nucleotide insertion increases the binding affinity to dNELF-E about 6-fold (K_d_; F-EMSA = 59±2 nM and FP = 82±1 nM) (Figure 3b,d and Table 1). Based on these experiments, we conclude that dNELF-E binds to TAR RNA, and that it targets an NBE-like motif within the loop region of TAR.

In light of this result, we wanted to clarify the specificity of the human form of NELF-E so we reexamined its interaction with HIV-1 TAR RNA. Because an NBE-like motif is present in TAR RNA (hereafter referred to as hNBE; human NELF-E binding element) and dNELF-E specifically targets the hNBE, it is plausible that hNELF-E actually binds this region of TAR, instead of the lower stem as previously reported. The wild-type TAR RNA sequence was first tested for binding with hNELF-E and found to bind with a higher affinity than previously reported (K_d_; F-EMSA = 300±20 nM and FP = 200±10 nM) (Figure 3c,d and Table 1) [25]. This may be due to amino acids outside of the RRM domain that contribute to the NELF-E RNA binding affinity.

To test if hNELF-E requires the hNBE in the loop region for its interaction, binding to the isolated dsRNA stem of TAR was examined. We generated a stem that lacks the hNBE (TAR-ΔhNBE) by annealing together two ssRNA sequences of TAR. No significant binding was detected with concentrations up to 2 µM hNELF-E protein (Figure 3c and Table 1). This was also observed for dNELF-E (Table 1). From this analysis, we conclude that hNELF-E, like dNELF-E, binds to the loop region of HIV-1 TAR, rather than the lower stem.

To compare the binding specificity of hNELF-E with dNELF-E, the affinity of hNELF-E was measured against TAR+A, which contains the NBE identified by SELEX in the loop region. This sequence does not bind significantly tighter, contrary to that observed with dNELF-E, but has a similar binding constant (K_d_; F-EMSA = 250±20 nM and FP = 250±20 nM) as unmodified TAR (Figure 3c,d and Table 1). This suggests that hNELF-E has a more flexible NBE specificity, CUGA_0–1_GGA, than dNELF-E.

### A non-conserved region within the RRM domain of *Drosophila* NELF-E is required for its differential specificity

**Figure 4:**
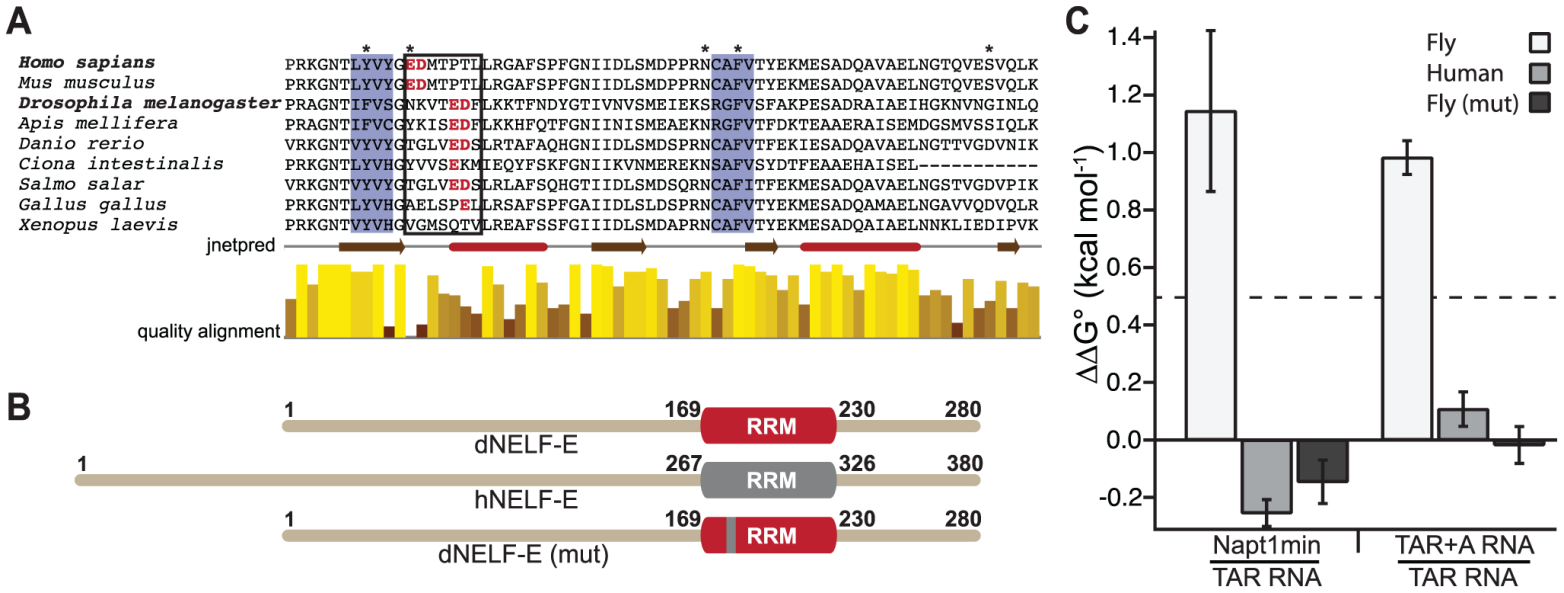
A humanized dNELF-E reveals an amino acid region that contributes to dNELF-E RNA recognition. (a) A sequence alignment of the RRM domain from a family of NELF-E proteins. Shaded in blue are the highly conserved ribonucleoprotein motifs RNP2 and RNP1. The boxed residues contain the seven amino acids that are mutated in the experiment shown in (b). Amino acids in red are the glutamate/aspartate residues that shift four positions toward the C-terminus in *Drosophila* and several other organisms. Asterisks represent positions that are thought to make RNA contacts [25]. Below the alignment is a secondary structure prediction obtained from jnetpred and a normalized quality alignment [55]–[57]. The brown arrows are beta sheets and the red tubes are alpha helices. (b) A summary of the mutagenesis performed on dNELF-E. The seven amino acid region boxed in (a) was humanized as illustrated in the domain structures. The grey region denotes the human RRM, while red signifies *Drosophila*. (c) The ΔΔG° for each NELF-E variant binding to either Napt1min and TAR RNA or TAR+A and TAR RNA. The K_d_ of each protein construct to its target was used to calculate the free energy (ΔG = −RT(lnK_d_)) from which the ΔΔG° values are derived. All experiments used full length protein constructs. Error bars represent the propagation of error derived from the standard deviations for indicated binding experiments. A ΔΔG° of 0.5 kcal mol^−1^ is shown by the dotted line.

To identify the region within the RRM that influences the differential specificity observed in dNELF-E, we aligned RRM domains from different species (Figure 4a) and noted a region that is not conserved between human and *Drosophila*, amino acids 269–275 in hNELF-E, as one of interest. hNELF-E has a glutamate residue in this region that has previously been defined as part of the RNA binding interface [24]. This residue and a proceeding aspartate are shifted four amino acids towards the C-terminus in *Drosophila* as well as in many other species examined and have a low alignment quality score relative to other positions in the RRM (Figure 4a).

To determine whether the amino acid shift observed in dNELF-E relative to hNELF-E accounts for the differences in RNA binding, we generated a humanized version of dNELF-E, dNELF-E(mut), that substitutes the seven amino acid region of dNELF-E with the human counterpart (Figure 4b). We then measured its binding to Napt1min, TAR, and TAR+A RNAs (Table 1). To assess the contribution that this region has on specificity, we used the observed binding constants (and those of dNELF-E and hNELF-E reported above) to calculate ΔΔG°, the difference between the standard binding free energies of the NELF-E variants to Napt1min and TAR (Figure 4c). A ΔΔG° measurement greater than 0.5 kcal mol^−1^ represents more than a two-fold change in binding affinity. Because dNELF-E binds much more tightly to Napt1min than to TAR RNA, the ΔΔG° is large (>1 kcal mol^−1^), while hNELF-E binds the two targets with similar affinities and has a small difference in binding free-energy (ΔΔG° = −0.25 kcal mol^−1^). The results for dNELF-E(mut) show that, like hNELF-E, it does not discriminate between the two targets (ΔΔG° = −0.15 kcal mol^−1^). This analysis was repeated comparing the binding of each NELF-E variant to TAR+A and TAR RNA (Figure 4c). A similar behavior was observed with these sequences as well. Based on these experiments, we conclude that the seven amino acid stretch tested in these experiments consists of residues that contribute to the binding specificity of *Drosophila* NELF-E. The reciprocal mutation made to hNELF-E does not, however, narrow the specificity of the hNELF-E (Figure S3). This implies that there are likely additional specificity determinants outside of the region tested that influence dNELF-E RNA recognition.

### The NBE is enriched in *Drosophila* promoter regions

**Figure 5:**
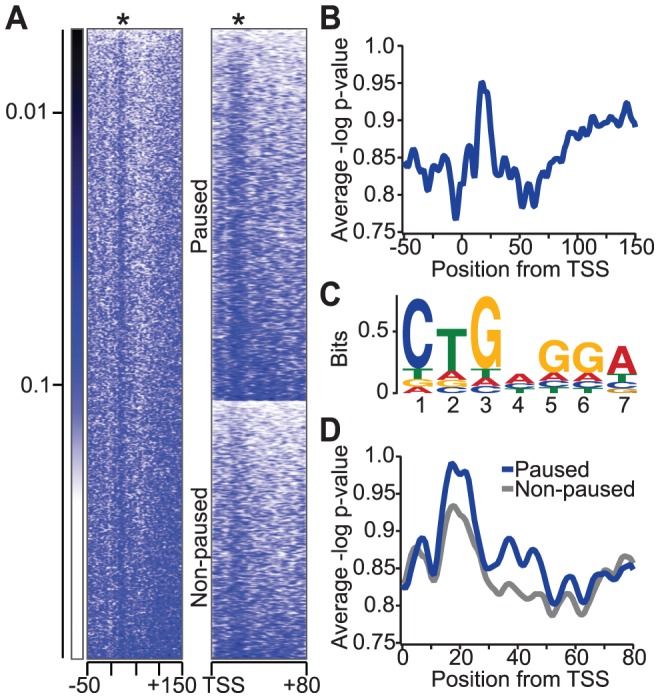
Relative enrichment of the NBE in *Drosophila* genomic regions near transcription start sites (TSSs). (a) Heat map of DNA sequence similarity to NBE in active *Drosophila* genes (n = 5471). Each row in the heat map represents a *Drosophila* gene from −50 to +150 base pairs from the TSS, and colors indicate the p-value of the sequence similarity index calculated from permutated 7-mer sequence scores. The asterisk indicates the position of NBE enrichment relative to the TSS. A heat map comparison of DNA sequence similarity for NBEs between paused (n = 3225) and non-paused (n = 2246) genes is shown to the right. Genes in each group are ordered by the strength of NBE similarity for comparison. (b) The average profile of the NBE similarity index in active genes. (c) A sequence logo representation of NBE-like sequences in active genes between +0 and +50 base pairs from the TSS for all genes. (d) The average profile of the NBE similarity index in paused and non-paused genes (p-value<7.2×10^−7^ by a Kolmogorov-Smirnov test or p-value<1.3×10^−5^ by a two-sample unequal variance t-test).

The NELF complex is highly enriched in promoter-proximal pause regions, and binding of the paused RNA transcript by co-localized NELF-E might support the ability of NELF to stabilize promoter-proximal paused Pol II [3], [14]. We hypothesized that the localization of NELF to these pause regions results, at least in part, from the enrichment of NBEs there. To test this, we searched for the NBE in *Drosophila* genomic regions near annotated transcription start sites (TSSs). The conserved seven-nucleotide NBE (CUGAGGA) that was characterized in this study (Figure 1a) was searched among all annotated *Drosophila* genes between −50 and +150 base pairs of TSSs (Figure 5a). Interestingly, we detect an enriched signal +20 to +30 base pairs downstream of the TSS, just upstream of the major Pol II pause site at +50 base pairs (Figure 5b, Figure S4) [2]. A sequence logo was generated using the identified motif in each sequence (Figure 5c). Interestingly, the observed motif is a more degenerate NBE than that identified by SELEX. We propose that weaker NELF-E binding sites might be tolerated or even preferred for some genes, allowing Pol II to release from the paused state more readily.

### A functional role for NBEs in transcription

If NELF-E's interaction with NBE-related sequences contributes to Pol II pausing, then these sequence elements should be more abundant in paused genes than in non-paused genes. Our group has previously mapped the genome-wide distribution of all transcriptionally engaged Pol II in *Drosophila* using GRO-seq, and more recently, at base-pair resolution using PRO-seq [2], [17]. Using these results, we found that there was a significant (two-sample unequal variance t-test p-value<1.3×10^−5^) increase of the NBE similarity index among paused genes compared to non-paused genes (Figure 5a and 5d). This result is consistent with the idea that NELF-E binding to nascent RNA transcripts contributes to pause formation and stabilization. In addition, enrichment of NBE-like sequences downstream of Pol II pause regions suggests that NELF-E might have a functional role downstream of the more prominent proximal-promoter pausing.

Transcription of HIV-1 provides a well-established model to assess the functionality of NBEs in Pol II promoter-proximal transcription regulation. As we have shown, hNELF-E binds specifically to the hNBE present within the stem loop of TAR (Figure 3), which clarifies the precise binding region for this known regulator of HIV-1 transcription. In agreement with this analysis, Feng and Holland previously reported that the loop region of TAR is essential for TAT trans-activation of an HIV-1 reporter [33]. They systematically mutagenized an HIV-1 reporter and demonstrated that the five-nucleotide element, CUGGG, in the stem-loop structure is a bona fide *cis*-regulatory element required for the activation of HIV-1 transcription. This pentanucleotide represents 5 of the 6 hNBE nucleotides. Moreover, this element is found in all three loops of a predicted HIV-2 TAR secondary structure [33].

The requirement of the NBE for HIV-1 transcription, as well as the presence of NBE-related sequences at the start of genes provoked us to analyze the binding of NELF-E to naturally transcribed RNAs. We combined the advantages of highly sensitive GRO-seq and our microcolumn based SELEX method to perform a SELEX experiment on nascent transcribed RNA. GRO-seq methodology was used to prepare a library of nascent RNAs (GRO-RNA) from transcriptionally engaged Pol II in *Drosophila* S2 cells. This allowed us to survey a pool of RNA sequences that are contextually relevant to NELF during transcription. One round of RAPID-SELEX (2 cycles with no amplification) [34] was performed using either dNELF-E as a target or a negative control with resin only. After high-throughput sequencing with the Illumina Hi-Seq platform, we searched for enrichment of NBE-like sequences (permitting 1 mutation) in the NELF-E selected pool and the resin only control pool using the pattern searching tool PatScan [35]. Since there are no amplification steps within the selection, enrichments were limited by the multiplicity of sequences within the initial GRO-RNA pool. Despite these limitations, there was still a significant enrichment of NBE-like sequences from NELF-E compared to the resin only control, as expected (p-value<2.2×10^−16^, Fisher's Exact Test) (Figure S5). This supports the hypothesis that NELF-E preferentially targets NBE sites in nascent RNA transcripts. Together, these data reveal that the NBE is enriched in contextually relevant regions and supports a biological role for NELF-E in promoter-proximal pausing.

## Discussion

RRM-domain proteins are known to have diverse modes of target recognition that can include a variety of specific RNA, DNA, and protein interactions [36]. Recent work has highlighted the role of these proteins in promoter-proximal pausing [37]. Our study here demonstrates that RRM-containing NELF-E is capable of binding to RNA with high affinity and sequence specificity (NBE: CUGAGGA(U) for *Drosophila*). NELF-E requires that the consensus be accessible in single-stranded RNA, and the binding can be enhanced with more complex secondary structures, such as the K-turn of Napt1min or the loop region of HIV-1 TAR RNA.

This work reveals that hNELF-E binds specifically to the HIV-1 TAR RNA stem loop that is closely related to the dNBE. These results have important implications for transcriptional regulation of HIV-1 by NELF and the P-TEFb-Tat complex. The hNBE overlaps the binding site for the P-TEFb subunit CycT1 and is adjacent to the TAR bulge region where Tat binds [32], [38], [39]. Instead of NELF-E binding to the lower stem as suggested previously [12], [19], our results indicate that NELF-E binds to the hNBE present in the loop to assist in establishment of a Pol II that is poised for transcription activation. After P-TEFb phosphorylation of NELF-E, we propose that the P-TEFb-Tat complex competes off NELF and releases Pol II into productive elongation. Further studies will unfold the complex interchange that occurs between these protein complexes to promote HIV-1 transcription, as well as a possible role for NELF in the establishment and maintenance of HIV-1 latency. The lower stem region of TAR does have a sequence that somewhat resembles the NBE (nucleotides 48 to 54 in Figure 3a); however, as we have shown, NELF-E does not bind to this double-stranded site with high affinity. For NELF to bind this site, the TAR stem would have to be melted to make the element accessible.

It is fitting that the NBE would be enriched in pause regions (+20 to +60 base pairs from the TSS) seeing that NELF plays a critical role in promoter-proximal pausing for many genes. Binding of NELF-E to this element might stabilize paused Poll II, working together with other pausing factors including DSIF [8], [21], the core promoter complex [1], [40], and GAGA factor [13]. It is possible that NELF-E binds RNA cooperatively with these factors, which could explain why the genomic NBE generated is more degenerate (Figure 5c) than the selected consensus sequence (Figure 1a). Additionally, the local proximity of the NELF complex with nascent RNA might be sufficient for an interaction and permit a weaker binding site.

An intriguing observation is the increased probability of NBE-like sequences >100 base pairs downstream of the TSS into the gene body (Figure 5b). This agrees with the Gilmour study, which detected a NELF interaction with longer transcripts (70 nucleotides) [21]. As described earlier, NELF is enriched in promoter-proximal regions and the observed binding location is, for many genes, broadly dispersed, even beyond the initial pause peak (maximal at +200 base pairs from the TSS) [3]. Perhaps there are multiple NELF-E interactions with the nascent RNA that assist in Pol II pausing as well as downstream RNA processes; and many genes might have “backup” NBE loci located downstream of the initial pause site. A possible role for these sites would be to provide a slow transition from the paused state into productive elongation before NELF dissociates from Pol II. Beyond the scope of this initial study, a detailed kinetic investigation of early elongation rates will help test this hypothesis. Alternatively, high affinity NBEs downstream might act as “deposit sites” to expel the NELF complex from paused Pol II and promote elongation.

In addition to its role in promoter-proximal pausing, evidence suggests that NELF may coordinate a number of mRNA processing steps during transcription [41]. Handa and coworkers have demonstrated that NELF interacts with the nuclear cap binding complex (CBC) to regulate the 3′ end processing of replication-dependent histone mRNAs. They also identify intranuclear focal accumulations of NELF, “NELF bodies,” that associate with RNA processing Cajal bodies and Cleavage bodies. Future studies will unveil the possible roles that NELF-E RNA binding has in other transcriptional and post-transcriptional regulatory mechanisms.

## Materials and Methods

### Protein expression and purification

Full length *Drosophila* and human NELF-E, and the RRM domain of dNELF-E (amino acid residues 147–247) were subcloned into pHIS-parallel1 to generate N-terminal hexahistindine-tagged recombinant proteins [42]. Mutated proteins were engineered using site-directed mutagenesis with primers that changed the corresponding codons for the 7 amino acids described in the text. Protein was expressed in BL21(DE3)-RIPL *E. coli* cells (Agilent Technologies). Liquid cultures were grown at 37°C and induced in mid-log phase with IPTG. Cultures were induced with either 1 mM IPTG at 37°C for 3 hours or 0.2 mM IPTG at 18°C overnight before collecting cells by centrifugation. Harvested cells were purified in batch according to the manufacturer's instructions for Ni-NTA Superflow (Qiagen) resins. Buffers used for the purification included lysis buffer (40 mM Tris-Cl, 300 mM NaCl, pH 8.0, 20 mM Imidazole, 10% glycerol, 5 mM 2-mercaptoethanol, EDTA-free protease inhibitor tablet (Roche Applied Science, 0.2 mg/ml lysozyme), wash buffer (lysis buffer with 200 mM NaCl), and elution buffer (wash buffer with 20% glycerol and 250 mM Imidazole). When necessary, eluted protein samples were subject to a mono Q column (GE Healthcare) for further purification as described elsewhere [43]. The quality of final protein products was analyzed by SDS-polyacrylamide gel electrophoresis. Purified samples were kept in elution buffer and small aliquots were flash frozen in liquid nitrogen and stored at −80°C.

### SELEX

A 120 nucleotide RNA library was generated as described [26]. The library was derived from a DNA template that consists of a 70 nucleotide randomized region flanked by two constant regions: 5′-AAGCTTCGTCAAGTCTGCAGTGAA-N70-GAATTCGTAGATGTGGATCCATTCCC-3′. This template allows for amplification and transcription using primers that are complementary to the constant regions and one primer encoding a T7 promoter. The starting RNA pool used in this selection had a complexity of >5×10^15^ unique molecules. Microcolumn SELEX was performed on dNELF-E and its RRM domain using a 20 µl column for each protein. A detailed method was previously described by Latulippe and Szeto et al. with some modifications [26]. The binding buffer used in this experiment consists of 10 mM HEPES-NaOH pH 7.5, 100 mM NaCl, 25 mM KCl, (5 mM MgCl_2_ for round 1 and 1 mM MgCl_2_ for each subsequent round), and 0.02% Tween-20. Wash buffer includes 20 mM Imidazole in the binding buffer.

### High-throughput sequencing and analysis of selected sequences

A purified PCR product from cycles 4 and 6 were re-amplified with barcoded primers and sequenced on the HiSeq 2000 (Illumina) sequencing platform using a standard single-end, 100 nucleotide sequencing protocol at the Cornell University Life Sciences Core Laboratory Center (http://cores.lifesciences.cornell.edu/brcinfo). Analysis of the sequencing data, which includes filtering and clustering analysis are described in detail by Latulippe and Szeto et al. [26]. The top 3000 unique DNA sequences in pool 6 obtained from the clustering analysis (see below) were subject to MEME [27] to derive a sequence logo for dNELF-E and its RRM domain. RNA secondary structure predictions were generated from the mfold web server [44].

### F-EMSA and fluorescence polarization

Fluorescence electrophoretic mobility shift (F-EMSA) and fluorescence polarization (FP) assays were performed as described previously [45]. The RNA sequences tested in this study were *in vitro* transcribed from synthetic DNA templates (Integrated DNA Technologies), PAGE purified, and eluted into DEPC treated 10 mM Tris-Cl pH 7.5. Napt1min includes an additional GC base pair on end to accommodate for the additional guanosine designed in the Napt1min template containing a T7 promoter. Purified RNA were then 3′-end labeled with fluorescein 5-thiosemicarbazide (Invitrogen) as described [31], [46]. (HIV-1 TAR-ΔhNBE RNA was prepared by annealing two synthesized RNA oligos (Integrated DNA Technologies) in annealing buffer (50 mM NaCl, 20 mM Tris pH 7.5, 1 mM EDTA). HIV-1 TAR-ΔhNBE was heat denatured (>60°C) at 1 µM concentration and cooled down to anneal before diluting samples for F-EMSA. All other RNAs were heated denatured in the F-EMSA binding buffer before adding protein. Binding reactions were prepared with 2 nM labeled RNA and varying concentrations of purified protein (from 0 to 2000 nM) in binding buffer (10 mM HEPES-NaOH pH 7.5, 100 mM NaCl, 25 mM KCl, 1 mM MgCl_2_, and 0.02% Tween-20, 0.01% IGEPAL CA-630, and 10 µg/ml yeast tRNA) to a final volume of 50 µl in black flat-bottom 96-well half-area microplates (Corning). It is recommended to use DEPC-treated water and SUPERase-In RNase inhibitor (Invitrogen) according to the manufacturers directions to prevent RNA degradation. Reactions were equilibrated for 1–2 hours before taking FP measurements on a Synergy H1 Microplate Reader (BioTek) with the appropriate filter cube for fluorescein (Ex: 485/20 Em: 528/20). After taking FP measurements, the same experiment was loaded on a pre-chilled 5% slab acrylamide gel (0.5X TBE) and electrophoresed at 4°C for approximately 1 hour and 10 minutes. Gels were imaged immediately on a Typhoon 9400 imager (GE Healthcare Life Sciences). The fluorescence intensity of bound and free RNA was measured with ImageQuant and the data was fit to a Hill equation in Igorpro software (Wavemetrics), which includes the Levenberg-Marquadt algorithm and statistical analysis tools [47].

### SELEX on GRO-RNA

Nuclei were isolated from non-heat-shocked *Drosophila* S2 cells as described previously [48]. Nuclear run-ons were performed using 2×10^7^ nuclei and GRO-seq libraries were prepared as in Core et al. [5], with the following specifications. Base hydrolysis of the nascent RNA was performed on ice for 20 min. 5′ and 3′ RNA adaptor sequences ligated to the run-on RNA were synthesized to match the constant regions of the N70 library [26]. cDNA synthesis was performed using a reverse oligo that anneals to the 3′ constant region (5′- AAGCTTCGTCAAGTCTGCAGTGAA-3′) and the library was amplified using this oligo and a forward oligo that recognizes the 5′ constant region and contains the T7 promoter (5′-GATAATACGACTCACTATAGGGAATGGATCCACAT CTACGA-3′), allowing the final library to utilize the same reagents that are used for preparation of SELEX pools between cycles. The final GRO-RNA library had an average size of ∼150–200 nucleotides including the constant regions.

Due to the relatively low complexity of the GRO-RNA library, a total of two selection cycles were completed using a method we call RAPID (RNA aptamer isolation via dual-cycles) which has been shown to significantly reduce the time and cost of isolating RNA aptamers and to improve enrichment rates, by systematically omitting amplification steps [34]. These RAPID selections were performed using 20 µl microcolumns loaded with 10 µM of full length dNELF-E, or with resin alone. The two selection cycles were completed in one round of RAPID, where the reverse transcription and amplification steps were omitted between cycles 1 and 2 to increase the specificity of the amplified and transcribed material that was used for downstream analysis. Purified PCR products from Pool 0 (initial GRO-RNA library) and Pools 2 were barcoded and sequenced as described below.

### Analysis of selected GRO-RNA

Control and experimental libraries were multiplexed and sequenced on an Illumina HiSeq 2000 instrument using a standard single-end, 100 nucleotide sequencing protocol at the Cornell University Life Sciences Core Laboratory Center (http://cores.lifesciences.cornell.edu/brcinfo). Following sequencing, reads were partitioned according to 5′-end barcode using the fastx_barcode_splitter.pl script from the FASTX-Toolkit v0.0.13.1 (http://hannonlab.cshl.edu/fastx_toolkit/). Barcodes were then trimmed using the fastx_trimmer utility from the FASTX-Toolkit. After trimming, the 5′ library preparation adapter was removed using the semi-global alignment and adapter removal utility cutadapt v1.1 (parameters: -g -e 0.20 -m 26 -O 18 –match-read-wildcards) [49]. Likewise, cutadapt was then used to remove the 3′ library preparation and sequencing adapters (parameters: -a -e 0.20 -O 2 -m 26). Given that the RNA library used for the SELEX experiments originated from NaOH-fragmented, nascently transcribed RNAs, we expected a heterogeneous distribution of sequencing read lengths. Therefore, we combined reads with and without a 3′ adapter into a single pool for all downstream analyses.

Trimmed sequences were then mapped to the *D. melanogaster* genome (assembly dm3) using 64-bit bowtie v0.12.7 allowing 2 possible mismatches and requiring unique alignment [50]–[52]. To account for fragmentation during the GRO library preparation, alignments were processed to obtain the genomic sequence beginning at the 5′ end of each mapping, and extended 100 bases downstream (the average length of the run-on RNA). These sequences were then analyzed using PatScan [35].

### DNA sequence similarity analysis of promoter regions

DNA sequence motifs were analyzed as described previously [2]. Briefly, DNA sequences were obtained from −50 to +150 base pair positions relative to the annotated TSS based on short capped nuclear RNA analysis in *Drosophila* [53]. For each position relative to the TSS, sequence similarity of the 7-mer to NBE was calculated from position weight matrix scores. The position weight matrix was built from the log-likelihood of an NBE consensus motif. p-values for the scores were calculated by comparing to 100,000 random permutated DNA sequence scores. The best matched 7-mer to the NBE consensus from +0 to +50 nucleotide positions relative to TSS were selected for every gene, and the base frequencies for each position were calculated. The base frequencies were used to generate the sequence logo as described previously [54]. For the comparison of paused and non-paused genes, gene lists were obtained from a previous study [2], and analyzed as described above. To test the statistical significance of the difference between paused and non-paused genes, the maximum of the NBE similarity score (–log_10_ p-value) within +10 to +30 base pair region for each gene was used as the test value, and the two groups were compared using a Kolmogorov-Smirnov test or two-sample unequal variance t-test. Sequence logos were generated as described previously [54] using an in-house script.

## Supporting Information

Figure S1 Secondary structure predictions of putative dNELF-E aptamers. The mfold web server was used to generate each structure; shown are the most thermodynamically stable predictions for each sequence analyzed. Nucleotides that make up the NBE are colored red. A sequence identification name is given below each putative aptamer.
(TIF)

Figure S2 dNELF-E binds to NBE containing aptamers. (a) Predicted secondary structure of Napt25 min with the NBE nucleotides shown in bold. (b) A representative F-EMSA of full length dNELF-E binding to Napt25 min. Below the gel is a plot of the fraction of bound Napt1min against protein concentration with a fit to the Hill equation. The equilibrium dissociation constant is shown in the graph for this individual experiment.
(TIF)

Figure S3 hNELF-E(mut) binds to HIV-1 TAR and HIV-1 TAR+A with a similar binding affinity. (top) A summary of the mutagenesis performed on hNELF-E. The seven amino acid region boxed as in Figure 4a was mutated as illustrated in the domain structures. The grey region denotes the human RRM, while red signifies *Drosophila*. (bottom) A representative plot of the fraction bound of either HIV-1 TAR (black line) or HIV-1 TAR+A (red line) RNA bound to hNELF-E(mut). A visual representation of each RNA tested is shown, with the inserted ‘A’ of TAR+A colored red.
(TIF)

Figure S4 The NBE enriches +20 to +30 nucleotides downstream the TSS. A boxplot of -log10(p-values) for the NBE near the TSS in Fig. 5b. The five positions are from 0±5 bp, 10±5 bp, 20±5 bp, 30±5 bp, and 40±5 bp from the TSS. The maximum -log10(p-values) within each range is the parameter of NELF binding in the region for active genes (n = 5471). The t-test p-values between adjacent groups are as follows: between 0 to 10 = 1.8×10^−22^, 10 to 20 = 1.1×10^−32^, 20 to 30 = 7.4×10^−43^, and 30 to 40 = 0.72 (not significant).
(TIF)

Figure S5 Enrichment of NBE-like sequences within genomic RNA. The abundance of sequences containing NBE-like motifs identified from a NELF-E SELEX compared to a resin only control. The bar graph shows the number of sequences containing an NBE where the variable position is indicated. Reads are plotted as multiplicity per million mapped reads (MPM).
(EPS)
