# Supplementary figures and images for: Defining NELF-E RNA Binding in HIV-1 and Promoter-Proximal Pause Regions
Source: PLoS Genet. 2014 Jan 16;10(1):e1004090. doi: 10.1371/journal.pgen.1004090 (PMC3894171; doi:10.1371/journal.pgen.1004090)

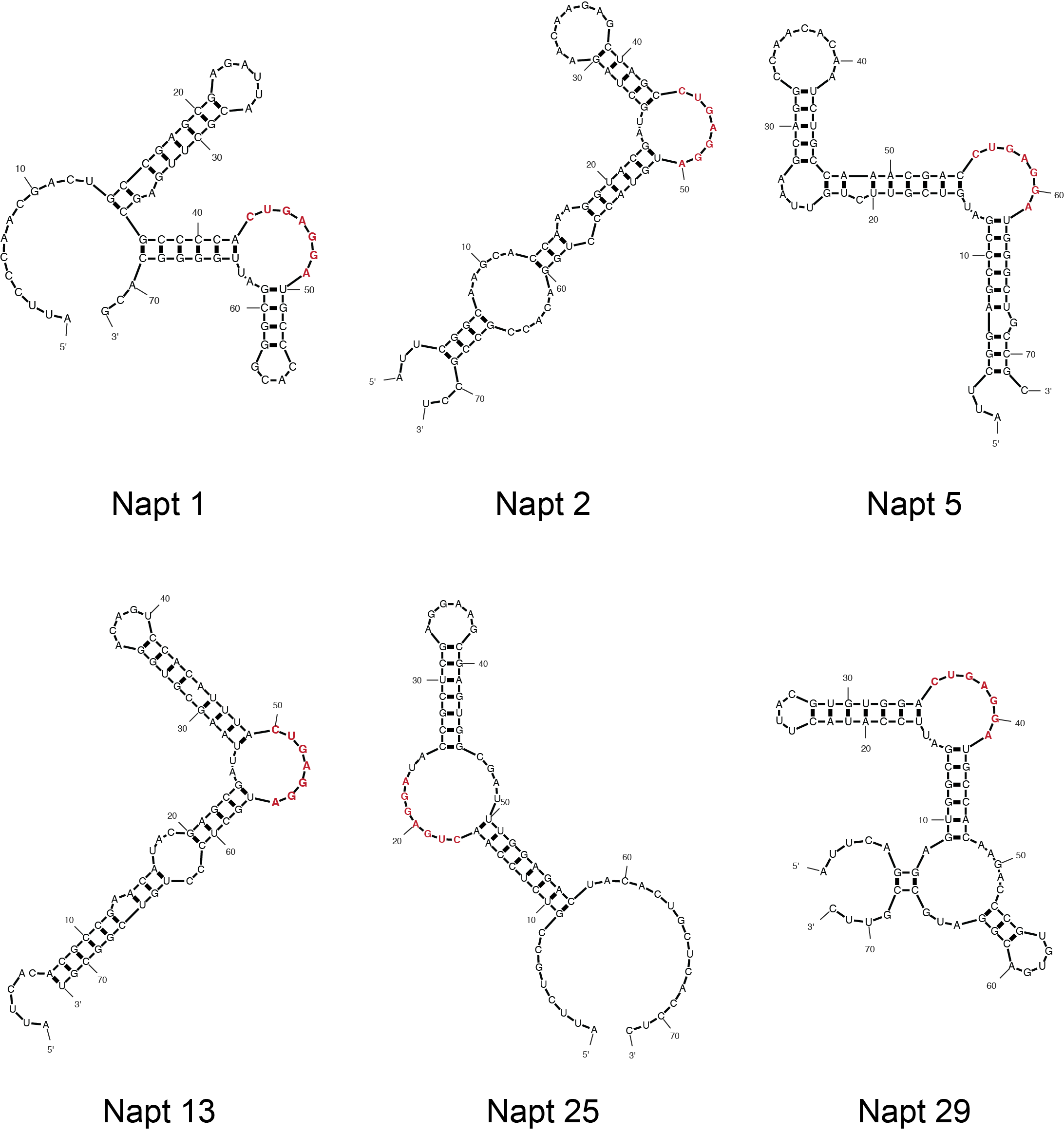

Supplement: Figure S1 — Secondary structure predictions of putative dNELF-E aptamers. The mfold web server was used to generate each structure; shown are the most thermodynamically stable predictions for each sequence analyzed. Nucleotides that make up the NBE are colored red. A sequence identification name is given below each putative aptamer. (TIF) [file pgen.1004090.s001.tif]

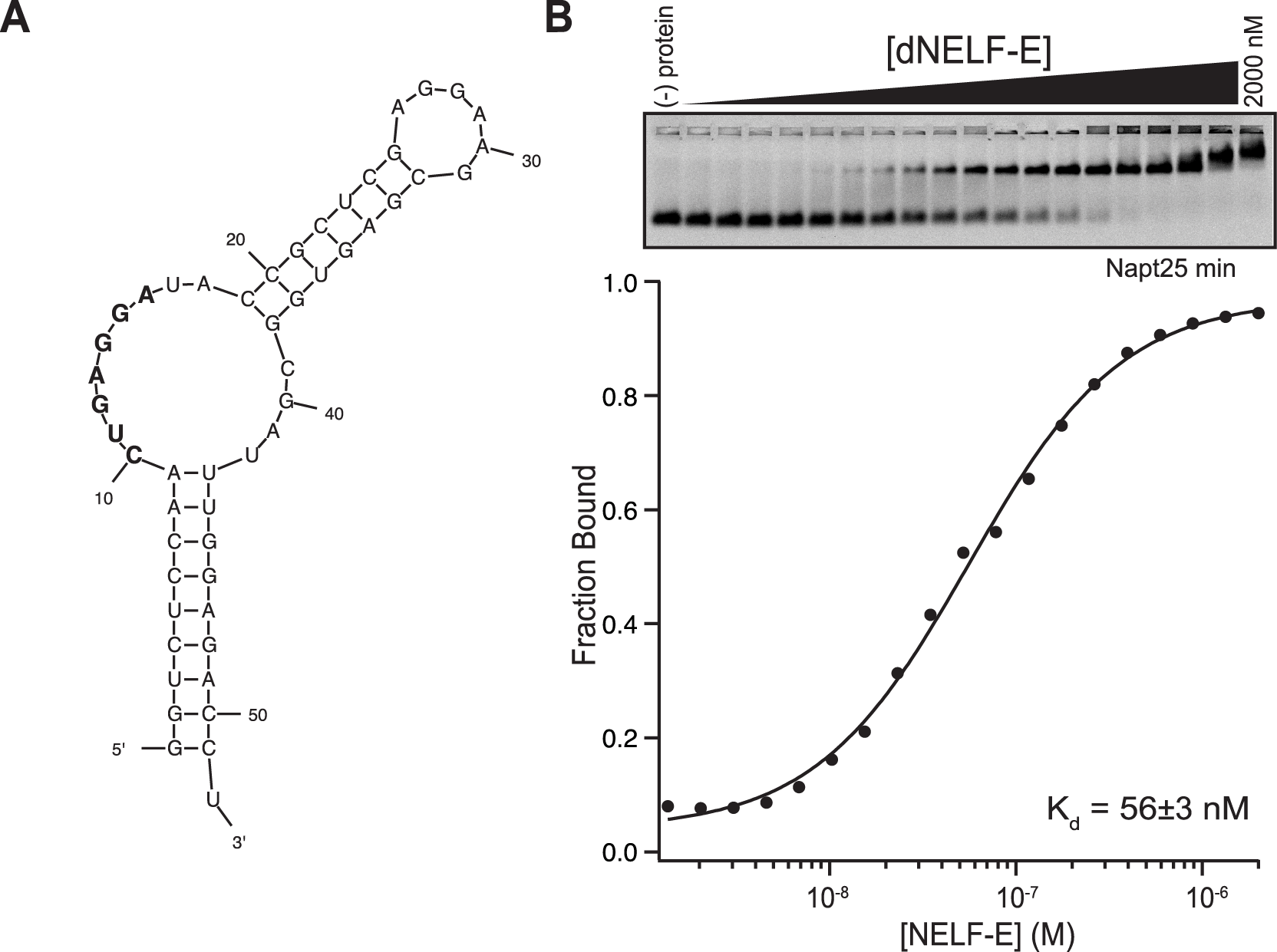

Supplement: Figure S2 — dNELF-E binds to NBE containing aptamers. (a) Predicted secondary structure of Napt25 min with the NBE nucleotides shown in bold. (b) A representative F-EMSA of full length dNELF-E binding to Napt25 min. Below the gel is a plot of the fraction of bound Napt1min against protein concentration with a fit to the Hill equation. The equilibrium dissociation constant is shown in the graph for this individual experiment. (TIF) [file pgen.1004090.s002.tif]

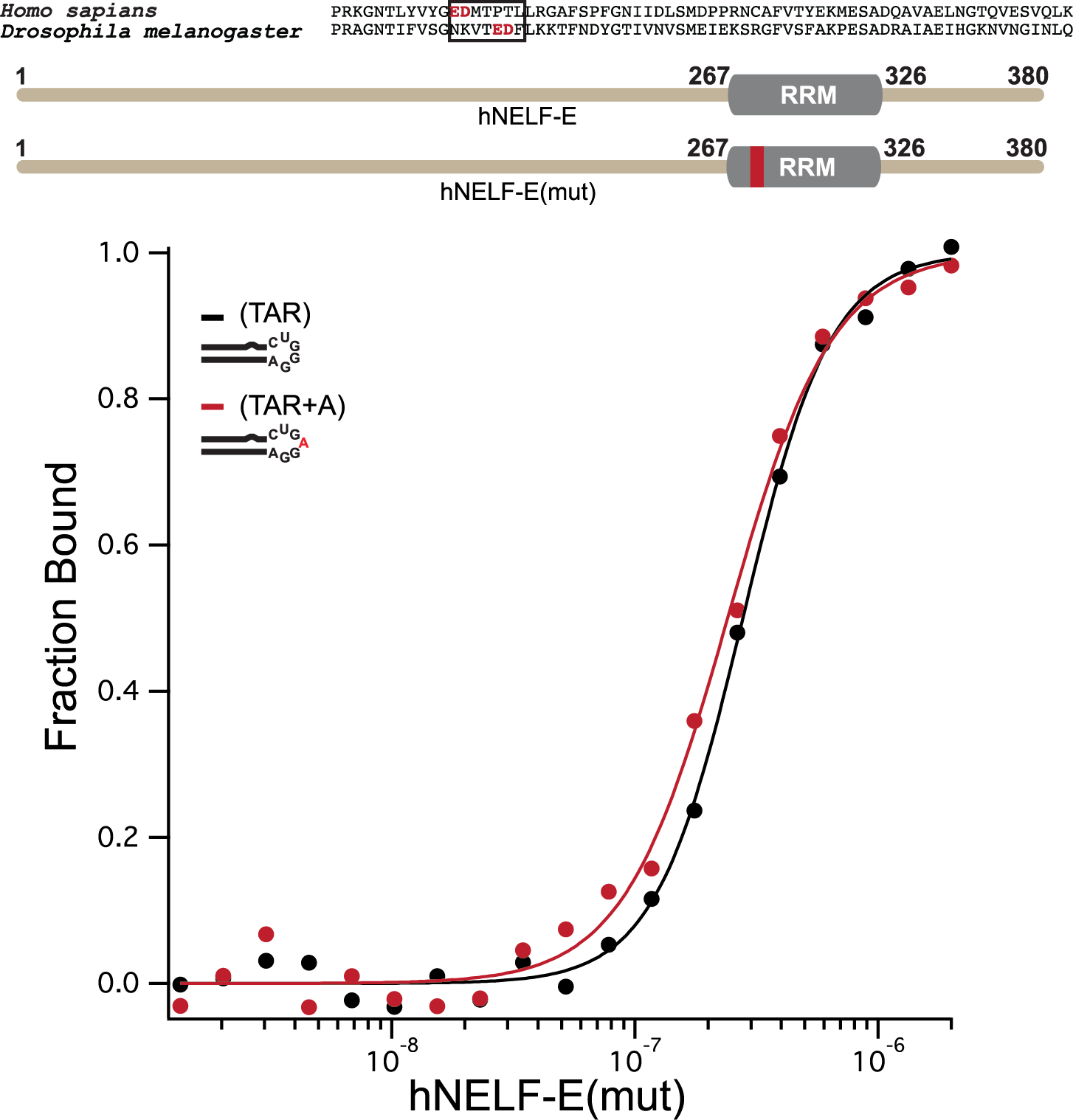

Supplement: Figure S3 — hNELF-E(mut) binds to HIV-1 TAR and HIV-1 TAR+A with a similar binding affinity. (top) A summary of the mutagenesis performed on hNELF-E. The seven amino acid region boxed as in Figure 4a was mutated as illustrated in the domain structures. The grey region denotes the human RRM, while red signifies Drosophila. (bottom) A representative plot of the fraction bound of either HIV-1 TAR (black line) or HIV-1 TAR+A (red line) RNA bound to hNELF-E(mut). A visual representation of each RNA tested is shown, with the inserted ‘A’ of TAR+A colored red. (TIF) [file pgen.1004090.s003.tif]

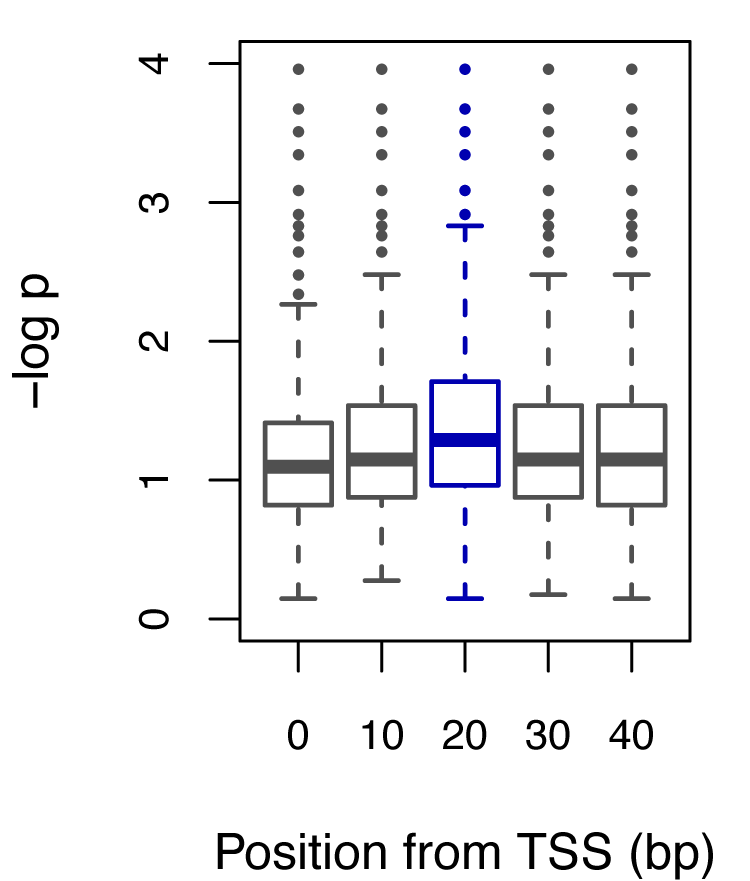

Supplement: Figure S4 — The NBE enriches +20 to +30 nucleotides downstream the TSS. A boxplot of -log10(p-values) for the NBE near the TSS in Fig. 5b. The five positions are from 0±5 bp, 10±5 bp, 20±5 bp, 30±5 bp, and 40±5 bp from the TSS. The maximum -log10(p-values) within each range is the parameter of NELF binding in the region for active genes (n = 5471). The t-test p-values between adjacent groups are as follows: between 0 to 10 = 1.8×10^−22^, 10 to 20 = 1.1×10^−32^, 20 to 30 = 7.4×10^−43^, and 30 to 40 = 0.72 (not significant). (TIF) [file pgen.1004090.s004.tif]
